# Post traumatic growth during COVID-19: unity in diversity

**DOI:** 10.1192/bjo.2021.69

**Published:** 2021-06-18

**Authors:** Meena Afzal Lakha, Anindya Bhowmik, Sneha Bisht, Suzani Shrestha, Kantappa Gajanan, Samir Shah

**Affiliations:** 1 Wrightington Wigan & Leigh NHS Foundation Trust; 2 London Northwest University Healthcare Trust; 3 Cambridge University Hospital NHS Foundation Trust; 4 Oxford University Hospital NHS Foundation Trust; 5 The Christie NHS Foundation Trust; 6 Priory Hospital Altrincham

## Abstract

**Aims:**

This poster reflects how the experience of staying with people of diverse nations and cultural background helped the stranded IMGs cope with this agony in a foreign land during an unprecedented tumultuous situation. The aim is to show that despite diversity among people, the hard times made them unite and overcome countless difficulties.

**Background:**

The COVID 19 pandemic has been a period of global health crisis and has exponentially affected mental health issues in the world population. In these difficult times, several International Medical Graduates (IMGs), who had come to the UK to attend their PLAB exams, were left stranded as the exams were postponed, flights cancelled and borders sealed. Faced with huge uncertainty their mental health was of great concern.

At this time the British Association of Physicians of Indian Origin (BAPIO) came forward to help this cohort of stranded doctors in terms of accommodation, finances, mental health support, preparation for exams to the extent of liaising with General Medical Council (GMC) and Home Office. The virtual support group provided a platform for IMGs from different nations and cultures to get in touch with each other helping overcome mental burden and stress.

The stories presented in the poster show how unity in diversity helped these young doctors deal with mental trauma amidst the Pandemic.

**Method:**

276 doctors from 27 countries were looked after by BAPIO. From those excerpts taken from 26 IMGs, personal narratives was used as a method for qualitative assessment.

The percentage of IMGs clearing their exams and getting jobs in the NHS has been used for quantitative assessment.

**Result:**

Qualitative: The personal narratives of the IMGs show how they were positively impacted by staying together albeit different nationalities and cultural background.

Quantitative: A total of 21 IMGs out of the 26 cleared their PLAB 2 exams and got registration under General Medical Council giving a percentage of 81.7%. 20 IMGs have successfully joined the NHS in various posts giving a job success rate of 95.2%.

**Conclusion:**

The experience of living and sharing housings with people from different nationalities, has increased appreciation and also prepared them to work in the NHS which has a diverse work force. This learning experience has been integral for all of us in shaping our life in the UK making everyone more compassionate.

